# Incidence of acute kidney injury after noncardiac surgery in patients receiving intraoperative dexmedetomidine: a retrospective study

**DOI:** 10.1016/j.bjao.2023.100136

**Published:** 2023-04-09

**Authors:** Manuel A. Paredes-Flores, Javier D. Lasala, Teresa Moon, Shreyas Bhavsar, Katherine Hagan, Sarah Huepenbecker, Nicolas P. Carram, Maria F. Ramirez, Kamal Maheswari, Lei Feng, Juan P. Cata

**Affiliations:** 1 Advocate Christ Medical Center Internal Medicine Residency, Oak Lawn, IL, USA; 2 Department of Anesthesiology and Perioperative Medicine, University of Texas MD Anderson Cancer Center, Houston, TX, USA; 3 Anesthesiology and Surgical Oncology Research Group, Houston, TX, USA; 4 Department of Gynecology and Reproductive Medicine, University of Texas MD Anderson Cancer Center, Houston, TX, USA; 5 Department of Anaesthesia, Hospital General de Agudos ‘Teodoro Alvarez’, Ciudad Autónoma de Buenos Aires, Buenos Aires, Argentina; 6 Department of General Anesthesia and Outcomes Research, Anesthesia Institute, Cleveland Clinic, Cleveland, OH, USA; 7 Department of Biostatistics, University of Texas MD Anderson Cancer Center, Houston, TX, USA

**Keywords:** acute kidney injury, dexmedetomidine, intraoperative dexmedetomidine, oncologic surgery

## Abstract

**Background:**

Postoperative acute kidney injury (AKI) is a common complication and is associated with increased hospital length of stay and 30 day all-cause mortality. Unfortunately, we have neither a defined strategy to prevent AKI nor an effective treatment. *In vitro*, animal, and human studies have suggested that dexmedetomidine may have a renoprotective effect. We conducted a retrospective cohort study to evaluate if intraoperative dexmedetomidine was associated with a reduced incidence of AKI.

**Methods:**

We collected data from 6625 patients who underwent major non-cardiothoracic cancer surgery. Before and after propensity score matching, we compared the incidence of postoperative AKI in patients who received intraoperative dexmedetomidine and those who did not. AKI was defined according to the Kidney Disease Improving Global Outcomes (creatinine alone values) criteria and calculated for postoperative Days 1, 2, and 3.

**Results:**

Twenty per cent (*n*=1301) of the patients received dexmedetomidine. The mean [standard deviation] administered dose was 78 [49.4] mcg. Patients treated with dexmedetomidine were matched to those who did not receive the drug. Patients receiving dexmedetomidine had a longer anaesthesia duration than the non-dexmedetomidine group. The incidence of AKI was not significantly different between the groups (dexmedetomidine 8% *vs* no dexmedetomidine 7%; *P*=0.333). The 30 day rates of infection, cardiovascular complications, or reoperation attributable to bleeding were higher in patients treated with dexmedetomidine. The 30 day mortality rate was not statistically different between the groups.

**Conclusions:**

The administration of dexmedetomidine during major non-cardiothoracic cancer surgery is not associated with a reduction in AKI within 72 h after surgery.

More than 17 million surgical procedures are performed annually in the USA and over 230 million worldwide.^1^, ^2^ Despite recent changes in clinical practice, one of the most common postoperative complications remains acute kidney injury (AKI), a sudden decrease in kidney function.^3^, ^4^ The incidence of AKI varies between 1% and 13% depending on several risk factors, including patients' comorbidities (i.e. age and diabetes mellitus) and type of surgery.^5^, ^6^, ^7^, ^8^, ^9^ AKI is associated with an increased length of hospital stay, 30 day all-cause mortality,^6^^,^^9^ and healthcare costs,^10^ which highlight the clinical relevance of AKI in the perioperative period.^7^ In patients undergoing non-cardiothoracic surgery, hypotension, ischaemia–reperfusion injury (IRI), and inflammation are amongst the more likely causes of AKI.^7^^,^^11^, ^12^, ^13^ However, there is neither an effective therapy nor a defined strategy to prevent postoperative AKI after non-cardiothoracic surgery.^3^, ^4^, ^5^, ^6^, ^7^

Dexmedetomidine is a highly selective alpha-2 adrenoceptor agonist. It has sedative, analgesic, and sympatholytic properties.^14^ Dexmedetomidine has several effects on renal function. It inhibits vasopressin at the collecting duct, causing diuresis, and preserves cortical blood flow by decreasing the renal cortical release of norepinephrine.^14^ Studies have also suggested that the renoprotective effect of dexmedetomidine against IRI is related to its antioxidant and anti-inflammatory properties.^11^, ^12^, ^13^, ^14^, ^15^, ^16^, ^17^ Specifically, dexmedetomidine reduces Toll-like receptor 4 and high-mobility group box-1 in tubular epithelial cells, which play a significant role in the inflammatory response during renal IRI.^11^^,^^18^ ^19^ Animal studies evaluating kidney damage at a cellular level found that pre- and post-treatment with dexmedetomidine significantly reduced tubular epithelial cell death.^11^ ^20^ ^21^ A pre-clinical study with rodent models showed that dexmedetomidine could affect cell malignancy by increasing tumour-cell retention and growth of metastases. However, the extrapolation of the findings to humans is uncertain.^22^ ^23^ Several clinical studies have investigated the impact of dexmedetomidine on postoperative AKI.^24^, ^25^, ^26^, ^27^ Two recent meta-analyses, including a mixed population of patients undergoing cardiac and noncardiac surgeries, demonstrated that dexmedetomidine reduces AKI.^28^^,^^29^

In summary, several *in vitro*, animal, and human studies have suggested that dexmedetomidine could have renoprotective effects.^11^^,^^16^ ^17^ ^19^ ^20^ ^25^^,^^29^ Based on this, our research question was, ‘Does intraoperative dexmedetomidine reduce AKI incidence after non-cardiothoracic cancer surgery?’ We hypothesised that a general anaesthetic technique, including intraoperative dexmedetomidine, was associated with a significant reduction in AKI incidence after non-cardiothoracic cancer surgery.

## Methods

We performed a retrospective cohort study to investigate the role of dexmedetomidine in preventing AKI after non-cardiothoracic surgery. Our Institutional Review Board (IRB#2021-0876) approved a retrospective cohort analysis of patients, who underwent major non-cardiothoracic surgery under general anaesthesia at the University of Texas MD Anderson Cancer Center from April 2016 to June 2021. The IRB waived the need for patients' written informed consent. Patients 18 yr and older undergoing major abdominal cancer surgery under general anaesthesia and with an expected hospital stay longer than 2 days were included. Patients were excluded if they did not have a preoperative serum creatinine or at least one postoperative serum creatinine during the first 3 days after surgery; if they had preoperative chronic kidney disease, defined as an estimated glomerular filtration rate (eGFR) of less than 60 ml min^−1^ (1.73 m)^−2^ for more than 3 months before the procedure; if they had emergency surgery; if they had an ASA physical status of >4; and patients undergoing thoracic procedures, head and neck surgery, urological procedures, craniotomies, integumentary procedures, herniorrhaphy, and transplantations. Patients requiring i.v. Contrast administration, reoperations during admission, and with unknown death/alive status 30 days after surgery were also excluded.

The intraoperative administration of dexmedetomidine as part of a general anaesthesia technique was our exposure variable. Patients were divided into two groups: those receiving intraoperative dexmedetomidine and those who did not. In our institution, dexmedetomidine is a component of an intraoperative anaesthesia management strategy geared towards enhancing the functional status of patients after surgery. Currently, the drug is given as an intraoperative i.v. Cnfusion (without a loading bolus dose) of 0.3 mcg kg^−1^ h^−1^ initiated after induction of general anaesthesia, titrated (0.1 mcg kg^−1^ h^−1^) against haemodynamic variables, and stopped at the time of surgical wound closure.

The primary outcome of this study was any degree of AKI. This was defined according to the Kidney Disease Improving Global Outcomes (KDIGO) criteria (creatinine values alone).^5^^,^^7^^,^^30^ Secondary outcomes, including a composite of 30 day complications (respiratory, surgical bleeding requiring surgery, neurological, gastrointestinal, cardiovascular, infectious, and renal), were obtained from the International Classification of Diseases, 10th Revision (ICD-10) codes. The 30 day mortality rate was estimated as the number of subjects who died within 30 days after surgery (all-cause mortality) divided by the total number of subjects in each group.

The following preoperative, intraoperative, and postoperative variables were collected from electronic medical records: age, BMI, ASA physical status classification, comorbidities, medications, serum creatinine, eGFR, electrolytes, complete blood count, and surgical procedure type. The General Surgery Acute Kidney Risk Index was utilised to estimate the individual risk of developing AKI.^6^ Intraoperative variables were haemodynamic variables; vasopressor use; urine output; fluid type, amount, and fluid balance; estimated blood loss; blood transfusion (type of product and number of units); and time elapsed from surgical incision to surgical closure. A serum creatinine within 30 days before surgery was used as a baseline concentration. Postoperative serum creatinine concentrations on Days 1, 2, and 3 were recorded to estimate AKI. Postoperative blood product use and postoperative complications within 30 days were also collected. Postoperative complications were extracted using ICD-10 codes after eligible patients were identified in the database. This paper adheres to the applicable Strengthening the Reporting of Observational Studies in Epidemiology guidelines.

### Statistical methods

Patient characteristics, treatment, and clinical outcomes were summarised through descriptive statistics. The Wilcoxon rank-sum test was used to compare location parameters of continuous distributions between patient groups. The χ2 test was used to evaluate the association between two categorical variables. We conducted a propensity score matching (PSM) analysis to adjust for selection bias in this observational study. The greedy 5→1 digit match algorithm was used to compare the baseline covariates so that the two groups (dexmedetomidine vs no dexmedetomidine) would have similar propensity scores for receiving dexmedetomidine. We included the following prognostic covariates in the multivariable logistic model to estimate the propensity scores: age at surgery, BMI, gender, ASA (1/2 vs 3/4), Charlson score (<5 vs ≥5), and operation type (open, laparoscopic, vs robotic). A multivariable logistic regression model was fitted to estimate the effects of important covariates on postoperative AKI. Statistical software SAS 9.4 (SAS, Cary, NC, USA) was used for all analyses.

## Results

**Fig 1:**
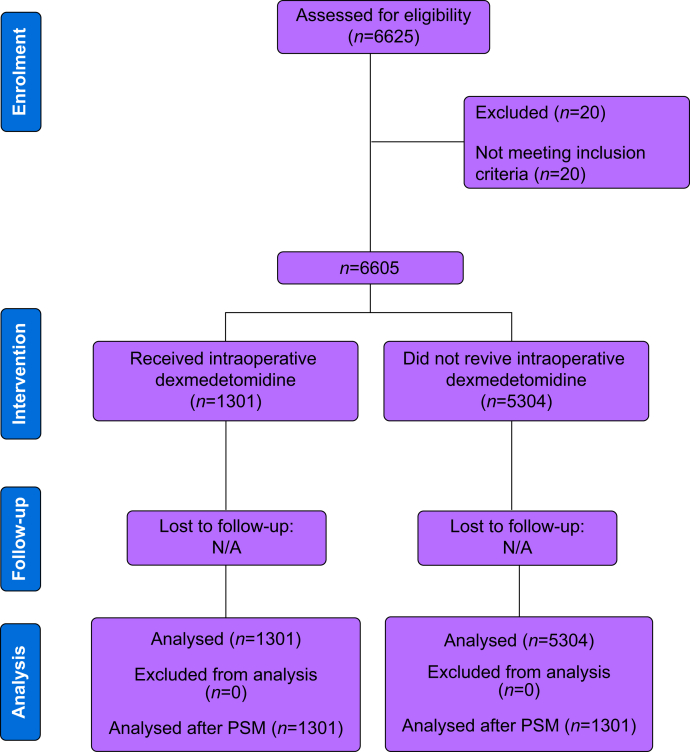
Consolidated Standards of Reporting Trials flow diagram. N/A, not applicable; PSM, propensity score matching.

A total of 6605 patients were included in the study (Fig 1). The overall rate of AKI on postoperative Day 1 (POD1), POD2, and POD3 was 4.13% (*n*=264), 6.32% (*n*=349), and 4.36% (*n*=195), respectively. Stage 1 AKI was the most frequently observed (POD1, *n*=213, 3.33%; POD2, *n*=263, 4.76%; and POD3, *n*=143, 3.19%), followed by Stages 2 (POD1, *n*=38, 0.59%; POD2, *n*=65, 1.18%; and POD3, *n*=30, 0.67%) and 3 (POD1, *n*=13, 0.2%; POD2, *n*=21, 0.38%; and POD3, *n*=22, 0.49%). One thousand three hundred and one (*n*=1301; 19.7%) patients received an infusion of dexmedetomidine during surgery. The mean (standard deviation) dose of dexmedetomidine administered was 78 [49.4] mcg. As shown in Table 1, several patient characteristics were unbalanced before PSM. After matching, the standardised differences for all covariates, including age at surgery, BMI, gender, ASA physical status (1–2 *vs* 3–4), Charlson score (<5 *vs* ≥5), and operation type (open, laparoscopic, *vs* robotic), were <4%, suggesting a substantial reduction of bias between the two groups. However, the operation site remained statistically different after matching.

**Table 1:** Patient characteristics and preoperative data. AKI, acute kidney injury; CCI, Charlson Comorbidity Index; Hb, haemoglobin; IQR, inter-quartile range; sd, standard deviation. Unknown metastatic status, *n*=84. Operation type: ‘open’ refers to a surgery in which the incision is large enough to let the surgeon see into the body. ‘Laparoscopic’ refers to surgery is done with the aid of a laparoscope. ‘Robotic’ refers to surgery that uses a computer-assisted mechanical device (robot).

Variable		Before matching			Post-matching			
		Dexmedetomidine		*P*-value	Dexmedetomidine			*P*-value
		No (*n*=5034)	Yes (*n*=1301)		No (*n*=1301)	Yes (*n*=1301)	Standardised difference (%)	
Age (yr)	Mean (sd)	58.04 (14.33)	54.11 (14.67)	<0.0001	53.95 (14.98)	54.11 (14.67)	1.12	
	Median (IQR)	59.99 (48.51–68.71)	55.2 (44.05–65.73)		55.19 (43.9–65.91)	55.2 (44.05–65.73)		
Gender	Male	3314 (62.5%)	816 (62.7%)	0.8728	808 (62.1%)	816 (62.7%)	1.27	
	Female	1990 (37.5%)	485 (37.3%)		493 (37.9%)	485 (37.3%)		
BMI	Mean (sd)	29.5 (10.4)	30.07 (12.25)	0.2994	30.30 (12.26)	30.07 (12.25)	1.91	
	Median (IQR)	27.87 (24.24–32.6)	28.12 (24.4–32.9)		28.25 (24.1–33.53)	28.12 (24.4–32.9)		
Race	White	4004 (75.5%)	982 (75.5%)	0.9941	989 (76%)	982 (75.5%)		0.315
	Non-White	1300 (24.5%)	319 (24.5%)		312 (24%)	319 (24.5%)		
ASA physical	1–2	411 (7.7%)	102 (7.8%)	0.9122	91 (7%)	102 (7.8%)	3.23	
status	2–4	4893 (92.3%)	1199 (92.2%)		1210 (93%)	1199 (92.2%)		
AKI	1	3670 (69.2%)	965 (74.2%)	0.0022	976 (75%)	965 (74.2%)		0.325
probability	2	1008 (19%)	219 (16.8%)		203 (15.6%)	219 (16.8%)		
score	3	487 (9.2%)	84 (6.5%)		99 (7.6%)	84 (6.5%)		
	4	124 (2.3%)	27 (2.1%)		21 (1.6%)	27 (2.1%)		
	5	15 (0.3%)	6 (0.5%)		2 (0.2%)	6 (0.5%)		
Metastatic	Unknown			0.0004				0.2127
disease	Non-metastatic	3697 (70.7%)	976 (75.7%)		944 (73.5%)	976 (75.7%)		
	Metastatic	1534 (29.3%)	314 (24.3%)		340 (26.5%)	314 (24.3%)		
CCI score	<5	2440 (46%)	724 (55.6%)	0.0001	720 (55.3%)	724 (55.6%)	0.62	
	≥5	2864 (54%)	577 (44.4%)		581 (44.7%)	577 (44.4%)		
Preoperative	No	807 (15.2%)	210 (16.1%)	0.4067	211 (16.2%)	210 (16.1%)		0.9575
NSAIDs	Yes	4497 (84.8%)	1091 (83.9%)		1090 (83.8%)	1091 (83.9%)		
Cardiovascular	No	2751 (51.9%)	595 (45.7%)	0.0001	589 (45.3%)	595 (45.7%)		0.8133
medications	Yes	2553 (48.1%)	706 (54.3%)		712 (54.7%)	706 (54.3%)		
Preoperative Hb (g dl^−1^)		12.66 (1.79) 12.8 (11.6–13.9)	12.82 (1.79) 12.9 (11.7–14.1)	0.028	12.73 (1.82) 12.9 (11.6–14)	12.82 (1.79) 12.9 (11.7–14.1)		0.473
Operation site	Gastrointestinal	1803 (34%)	387 (29.7%)	0.0001	404 (31.1%)	387 (29.7%)		0.0006
	Orthopaedic	1111 (20.9%)	197 (15.1%)		249 (19.1%)	197 (15.1%)		
	Gynaecological	568 (10.7%)	176 (13.5%)		149 (11.5%)	176 (13.5%)		
	Breast surgery	59 (1.1%)	25 (1.9%)		16 (1.2%)	25 (1.9%)		
	Neurosurgery	293 (5.5%)	50 (3.8%)		79 (6.1%)	50 (3.8%)		
	Other	1470 (27.7%)	466 (35.8%)		404 (31.1%)	466 (35.8%)		
Operation	Open	4029 (76%)	1016 (78.1%)	0.0001	1023 (78.6%)	1016 (78.1%)	2.69	
type	Laparoscopic	1039 (19.6%)	197 (15.1%)		203 (15.6%)	197 (15.1%)		
	Robotic	236 (4.4%)	88 (6.8%)		75 (5.8%)	88 (6.8%)		

Table 2 shows intraoperative variables in patients treated with and without dexmedetomidine. After matching, patients in the dexmedetomidine group (median [inter-quartile range] 378 [287–513] min) had a longer duration of anaesthesia than those in the non-dexmedetomidine group (322 [240–440] min). The general anaesthetic technique was also different between patients treated with dexmedetomidine *vs* no dexmedetomidine. The propofol-based TIVA rate was higher in patients treated with dexmedetomidine (*n*=208; 16%) than in the non-dexmedetomidine group (*n*=47; 3.6%). Patients receiving dexmedetomidine were more likely to receive adjuvant i.v. Anaesthetics, such as lidocaine (*n*=66, 5.1% *vs* no dexmedetomidine, *n*=31, 2.4%) or ketamine (*n*=607, 46.4% *vs* no dexmedetomidine, *n*=3, 0.2%). The rate of i.v. Acetaminophen use was also higher in patients treated with dexmedetomidine (*n*=801; 61.6%) than in the non-dexmedetomidine group (*n*=732; 56.3%). More patients were not given opioids intraoperatively in the dexmedetomidine group (*n*=35, 2.7% *vs* non-dexmedetomidine, *n*=11, 0.8%). Of those who received opioids, the mean intraoperative morphine equivalent dose was statistically different between treatment groups; however, the difference was not clinically relevant (Table 2).

**Table 2:** Intraoperative variables in patients treated with and without dexmedetomidine. FFP, fresh frozen plasma; Intraop., intraoperative; IQR, inter-quartile range; MEDD, morphine equivalent daily dose; PRBCs, packed red blood cells; VA, volatile anaesthesia.

Variable		Before matching			After matching		
		Dexmedetomidine, no (*n*=5304)	Dexmedetomidine, yes (*n*=1301)	*P*-value	Dexmedetomidine, no (*n*=1301)	Dexmedetomidine, yes (*n*=1301)	*P*-value
Anaesthesia				<0.0001			<0.0001
duration (min)	Median (IQR)	318 (232.5–439)	378 (287–513)		322 (240–440)	378 (287–513)	
Anaesthesia	VA	5112 (96.4%)	1093 (84%)	<0.0001	1254 (96.4%)	1093 (84%)	<0.0001
technique	TIVA	192 (3.6%)	208 (16%)		47 (3.6%)	208 (16%)	
Infusion i.v.	No	5290 (99.7%)	697 (53.6%)	<0.0001	1298 (99.8%)	697 (53.6%)	<0.0001
ketamine	Yes	14 (0.3%)	604 (46.4%)		3 (0.2%)	604 (46.4%)	
Infusion i.v.	No	5159 (97.3%)	1235 (94.9%)	<0.0001	1270 (97.6%)	1235 (94.9%)	0.0003
lidocaine	Yes	145 (2.7%)	66 (5.1%)		31 (2.4%)	66 (5.1%)	
Intraoperative	No	2359 (44.5%)	500 (38.4%)	0.0001	569 (43.7%)	500 (38.4%)	0.0060
acetaminophen	Yes	2945 (55.5%)	801 (61.6%)		732 (56.3%)	801 (61.6%)	
Intraoperative	Yes	5262 (99.2%)	1266 (97.3%)	<0.0001	1290 (99.2%)	1266 (97.3%)	0.0004
opioids	No	42 (0.8%)	35 (2.7%)		11 (0.8%)	35 (2.7%)	
Intraoperative				0.416			0.001
MEDD (mg)	Median (IQR)	4.5 (2–7.5)	4.5 (2–7.5)		5.5 (2.5–7.5)	5.5 (2.5–7.5)	
Intraoperative	No	1275 (24%)	286 (22%)	0.1179	355 (27.3%)	286 (22%)	0.0017
vasopressors	Yes	4029 (76%)	1015 (78%)		946 (72.7%)	1015 (78%)	
Ephedrine				0.1376			0.859
dose (mg)	Median (IQR)	15 (10–15)	15 (10–15)		15 (10–25)	15 (10–25)	
Phenylephrine dose (mg)	Median (IQR)	300 (200–700)	300 (150–700)	0.857	300 (150–600)	300 (150–700)	0.439
Intraoperative crystalloids (ml)	Median (IQR)	1300 (1000–1800)	1550 (1100–2100)	<0.0001	1300 (1000–1950)	1550 (1100–2100)	<0.0001
Intraoperative	No	2272 (42.8%)	394 (30.3%)	<0.0001	533 (41%)	394 (30.3%)	<0.0001
albumin	Yes	3032 (57.2%)	907 (69.7%)		768 (59%)	907 (69.7%)	
Intraoperative				<0.0001			<0.0001
albumin (ml)	Median (IQR)	250 (0–750)	500 (0–1000)		500 (0–750)	500 (0–1000)	
Estimated blood loss (ml)	Median (IQR)	150 (50–350)	200 (100–400)	<0.0001	150 (50–350)	200 (100–400)	<0.0001
Intraoperative	No	4721 (89%)	1144 (87.9%)	0.2702	1156 (88.9%)	1144 (87.9%)	0.4627
PRBCs	Yes	583 (11%)	157 (12.1%)		145 (11.1%)	157 (12.1%)	
Intraoperative	No	5247 (98.9%)	1284 (98.7%)	0.4761	1284 (98.7%)	1284 (98.7%)	1.000
platelets	Yes	57 (1.1%)	17 (1.3%)		17 (1.3%)	17 (1.3%)	
Intraoperative	No	5166 (97.4%)	1255 (96.5%)	0.0666	1262 (97%)	1255 (96.5%)	0.4401
FFP	Yes	138 (2.6%)	46 (3.5%)		39 (3%)	46 (3.5%)	
Intraoperative	No	5299 (99.9%)	1296 (99.6%)	0.0159	1299 (99.8%)	1296 (99.6%)	0.2562
cryoprecipitate	Yes	5 (0.1%)	5 (0.4%)		2 (0.2%)	5 (0.4%)	
Urinary				<0.0001			<0.0001
output (ml)	Median (IQR)	325 (200–515)	400 (250–632.5)		325 (200–525)	400 (250–632.5)	

**Fig 2:**
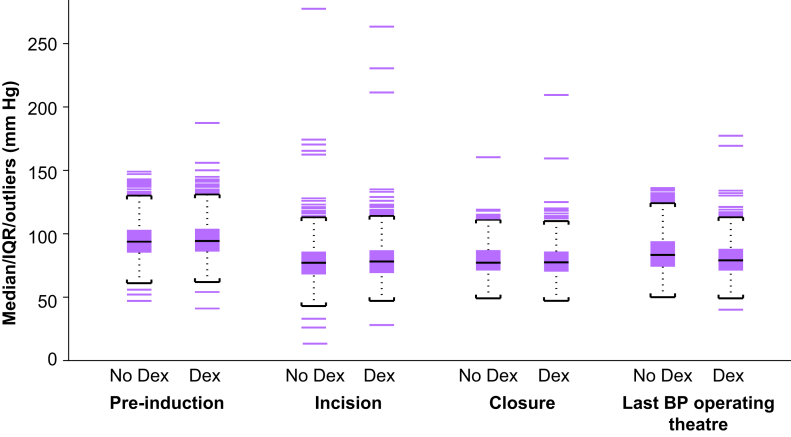
MAPs in patients treated with and without dexmedetomidine. Dex, dexmedetomidine; IQR, inter-quartile range.

Analysis of blood pressure (Fig 2) and heart rate before induction of general anaesthesia, at the time of surgical incision, at wound closure, and before leaving the operating theatre showed statistical but not clinically relevant differences between the groups. Whilst the rate of patients requiring vasopressors (i.e. phenylephrine or ephedrine) intraoperatively was higher in the dexmedetomidine group (*n*=1015; 78%) than in the non-dexmedetomidine group (*n*=946; 72.7%), the doses of phenylephrine or ephedrine administered were not statistically different (Table 2). Intraoperative fluid administration also differed statistically between the groups. Those who received dexmedetomidine were administered a significantly larger volume of crystalloids (dexmedetomidine 1550 [1100–2100] ml *vs* non-dexmedetomidine 1300 [1000–1950; *P*<0.0001]) and albumin (dexmedetomidine 500 [0–1000] ml *vs* non-dexmedetomidine 500 [0–750] ml; *P*<0.0001) than those in the non-dexmedetomidine group (Table 2). Blood loss was slightly higher in patients who received dexmedetomidine (200 [100–400] ml) than those not treated with the drug (150 [50–350] ml; *P*<0.0001). Lastly, patients in the dexmedetomidine group had a mean higher urinary output (400 [250–632.5] ml) than those in the non-dexmedetomidine group (325 [200–525] ml; *P*<0.0001).

### Postoperative AKI and complications

**Fig 3:**
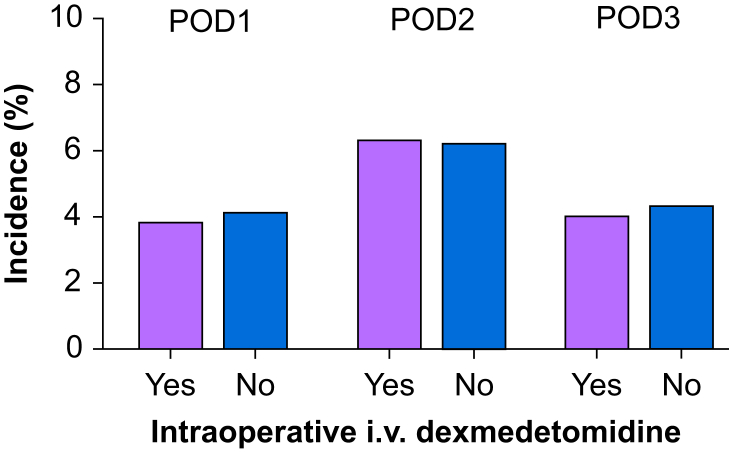
Bar graph illustrates the daily postoperative incidence of AKI (any stage) after propensity score matching. The i.v. administration of dexmedetomidine (open bars) was not associated with reducing AKI. AKI, acute kidney injury; POD, postoperative day.

As shown in Fig 3, the daily incidence of postoperative AKI was not significantly different between the groups. AKI peaked on POD2 when the rate was 6.4% and 5.8% in patients treated with and without dexmedetomidine added to the general anaesthesia regimen, respectively. Amongst the subgroup of matched patients in the cohort (41%), the observed risk of early AKI and severity did not differ between patients who received and did not receive dexmedetomidine as part of the general anaesthesia regimen (Table 3). With the adjustment of BMI (>25 *vs* ≤25), ASA (3–4 *vs* 1–2), AKI probability score, operation type, intraoperative use of vasopressors, and administration of blood products, the association between the use of dexmedetomidine as part of the general anaesthetic technique and postoperative AKI was not significant (odds ratio 1.12; 95% confidence interval [CI]: 0.831–1.514; *P*=0.4535; Table 4). The analysis showed that an AKI probability score >1, open surgery, intraoperative blood transfusions, and the administration of vasopressors were independent risk factors of AKI within 72 h after surgery (Table 4).

**Table 3:** Postoperative clinical outcomes before and after PSM. AKI, acute kidney injury; FFP, fresh frozen plasma; POD, postoperative day; PRBC, packed red blood cell; PSM, propensity score matching.

Variable		Before PSM			After PSM		
		Dexmedetomidine, no (*n*=5304)	Dexmedetomidine, yes (*n*=1301)	*P*-value	Dexmedetomidine, no (*n*=1301)	Dexmedetomidine, yes (*n*=1301)	*P*-value
POD1				0.5720			0.9899
AKI Stage 1		176 (3.4%)	37 (2.9%)		39 (3.1%)	37 (2.9%)	
AKI Stage 2		28 (0.5%)	10 (0.8%)		9 (0.7%)	10 (0.8%)	
AKI Stage 3		11 (0.2%)	2 (0.2%)		2 (0.2%)	2 (0.2%)	
POD2				0.4396			0.3826
AKI Stage 1		207 (4.7%)	56 (4.8%)		47 (4.4%)	56 (4.8%)	
AKI Stage 2		48 (1.1%)	17 (1.5%)		10 (0.9%)	17 (1.5%)	
AKI Stage 3		19 (0.4%)	2 (0.2%)		5 (0.5%)	2 (0.2%)	
POD3				0.4585			0.8150
AKI Stage 1		114 (3.2%)	29 (3%)		26 (3%)	29 (3%)	
AKI Stage 2		22 (0.6%)	8 (0.8%)		8 (0.9%)	8 (0.8%)	
AKI Stage 3		20 (0.6%)	2 (0.2%)		4 (0.5%)	2 (0.2%)	
Postoperative PRBC	No	4554 (85.9%)	1102 (84.7%)	0.2869	1121 (86.2%)	1102 (84.7%)	0.291
transfusion	Yes	750 (14.1%)	199 (15.3%)		180 (13.8%)	199 (15.3%)	
Postoperative platelet	No	5267 (99.3%)	1285 (98.8%)	0.0538	1292 (99.3%)	1285 (98.8%)	0.159
transfusion	Yes	37 (0.7%)	16 (1.2%)		9 (0.7%)	16 (1.2%)	
Postoperative FFP	No	5257 (99.1%)	1271 (97.7%)	<0.0001	1290 (99.2%)	1271 (97.7%)	0.002
transfusion	Yes	47 (0.9%)	30 (2.3%)		11 (0.8%)	30 (2.3%)	
Postoperative cryoprecipitate	No	5298 (99.9%)	1294 (99.5%)	0.0019	1300 (99.9%)	1294 (99.5%)	0.033
transfusion	Yes	6 (0.1%)	7 (0.5%)		1 (0.1%)	7 (0.5%)	
Postoperative 30 day	No	4423 (83.4%)	1029 (79.1%)	0.0003	1085 (83.4%)	1029 (79.1%)	0.0049
overall complications	Yes	881 (16.6%)	272 (20.9%)		216 (16.6%)	272 (20.9%)	
Postoperative 30 day	No	5058 (95.4%)	1219 (93.7%)	0.0132	1246 (95.8%)	1219 (93.7%)	0.0178
cardiovascular complications	Yes	246 (4.6%)	82 (6.3%)		55 (4.2%)	82 (6.3%)	
Postoperative 30 days	No	5180 (97.7%)	1268 (97.5%)	0.6734	1266 (97.3%)	1268 (97.5%)	0.8059
respiratory complications	Yes	124 (2.3%)	33 (2.5%)		35 (2.7%)	33 (2.5%)	
Postoperative 30 day	No	5094 (96%)	1248 (95.9%)	0.8499	1246 (95.8%)	1248 (95.9%)	0.8442
neurological complications	Yes	210 (4%)	53 (4.1%)		55 (4.2%)	53 (4.1%)	
Postoperative 30 day	No	5087 (95.9%)	1222 (93.9%)	0.0020	1247 (95.8%)	1222 (93.9%)	0.0261
infectious complications	Yes	217 (4.1%)	79 (6.1%)		54 (4.2%)	79 (6.1%)	
Postoperative 30 day	No	5000 (94.3%)	1199 (92.2%)	0.0045	1223 (94%)	1199 (92.2%)	0.0637
gastrointestinal complications	Yes	304 (5.7%)	102 (7.8%)		78 (6%)	102 (7.8%)	
Postoperative 30 day	No	5269 (99.3%)	1288 (99%)	0.1966	1291 (99.2%)	1288 (99%)	0.5298
renal complications	Yes	35 (0.7%)	13 (1%)		10 (0.8%)	13 (1%)	
Postoperative 30 day	No	5294 (99.8%)	1296 (99.6%)	0.1837	1296 (99.6%)	1296 (99.6%)	1.000
Multi-organ failure	Yes	10 (0.2%)	5 (0.4%)		5 (0.4%)	5 (0.4%)	
Postoperative 30 day	No	5244 (98.9%)	1276 (98.1%)	0.0234	1288 (99%)	1276 (98.1%)	0.0499
bleeding with reoperation	Yes	60 (1.1%)	25 (1.9%)		13 (1%)	25 (1.9%)	
Postoperative 30 day	No	5279 (99.5%)	1297 (99.7%)	0.4230	1295 (99.5%)	1297 (99.7%)	0.5263
mortality	Yes	25 (0.5%)	4 (0.3%)		6 (0.5%)	4 (0.3%)

**Table 4:** Multivariate analysis to estimate the effects of important covariates on postoperative AKI. AKI, acute kidney injury; CI, confidence interval; OR, odds ratio.

OR estimates and wald CIs				
Effect	*P*-value	OR estimate	95% CI	
BMI (≥25 *vs* <25)	0.0060	1.683	1.161	2.441
ASA 3–4 *vs* 1–2	0.0529	2.456	0.989	6.102
AKI probability score (2 *vs* 1)	0.0124	1.608	1.108	2.333
AKI probability score (3 *vs* 1)	0.0829	1.593	0.941	2.695
AKI probability score (4 *vs* 1)	0.0450	2.338	1.019	5.362
AKI probability score (5 *vs* 1)	0.2286	2.806	0.523	15.050
Open *vs* robotic surgery	0.0343	2.987	1.084	8.226
Laparoscopic *vs* robotic surgery	0.2454	1.909	0.641	5.684
Dexmedetomidine use (yes *vs* no)	0.4535	1.122	0.831	1.514
Intraoperative vasopressors (no *vs* yes)	0.0242	0.616	0.405	0.939
Intraoperative blood products (yes *vs* no)	<0.0001	3.104	2.198	4.384

We observed that adding dexmedetomidine to the general anaesthesia technique was associated with an increase in other postoperative complications. Specifically, we found that 30 day overall complications rate (dexmedetomidine, *n*=272, 20.9% *vs* no dexmedetomidine, *n*=216, 16.6%; *P*=0.017), infections (dexmedetomidine, *n*=79, 6.1% *vs* no dexmedetomidine, *n*=54, 4.2%; *P*=0.026), cardiovascular complications (dexmedetomidine, *n*=82, 6.3% *vs* no dexmedetomidine, *n*=55, 4.2%; *P*=0.017), and 30 day bleeding requiring surgery (dexmedetomidine, *n*=25, 1.9% *vs* no dexmedetomidine, *n*=13, 1%; *P*=0.049) were significantly higher in the dexmedetomidine group than in the non-dexmedetomidine group. The 30 day mortality rate was not significantly different between the groups.

## Discussion

Acute kidney injury is a frequent postoperative complication after major noncardiac cancer surgery.^31^ Our study demonstrates that an intraoperative infusion of dexmedetomidine was not associated with a reduction in AKI rates or 30 day renal complications after major noncardiac cancer surgery. The fact that our study failed to show any differences between the incidence of AKI in dexmedetomidine *vs* non-dexmedetomidine groups has several possible explanations. The lack of renoprotective effects in our study has several possible reasons. First, the lack of a bolus dose at the initiation of the intraoperative infusion of dexmedetomidine may have limited the concentration of the drug in the kidney during surgery, and thus its full potential for renal protection. However, we did observe dexmedetomidine's diuretic effect, which is thought to be associated with renoprotection. Next, dexmedetomidine is frequently associated with hypotension, and we did notice a higher rate of vasopressor (phenylephrine and ephedrine) use in patients who received dexmedetomidine as part of the general anaesthesia technique than in those who did not receive the drug. However, the duration of hypotension cannot be readily deduced from our data set, so its effect on renal protection is difficult to speculate. Second, AKI peaked on POD2 in both groups of patients. It has been reported that the elimination half-life of dexmedetomidine in non-critically ill patients is 2–2.5 h.^32^ Therefore, postoperative renal injury may be caused by hypotension that occurs on the ward, negating the renoprotective effect of intraoperative dexmedetomidine.^33^ Finally, there could be a true negative relationship, and dexmedetomidine has no effect on kidney injury incidence; this should be evaluated by future RCTs.

A meta-analysis and trial sequential analysis of nine RCTs with 1308 patients found robust evidence that dexmedetomidine significantly reduced AKI incidence after cardiac surgery (risk ratio 0.60; 95% CI: 0.41–0.87; *P*=0.008).^29^ Results from two recent meta-analyses that included a mixed population of cardiac and noncardiac, cancer, and non-cancer surgeries have also demonstrated that dexmedetomidine reduces AKI in critically ill patients.^28^^,^^29^ Previous studies have investigated the association between the intraoperative use of dexmedetomidine as part of the general anaesthesia technique and postoperative AKI in cancer surgery.^34^^,^^35^ In a pilot randomised controlled study (*n*=89) in which placebo or dexmedetomidine was given intraoperatively to patients undergoing laparoscopic radical prostatectomy for cancer, the incidence of AKI was reduced from 13.3% (dexmedetomidine) to 4.5% (placebo). However, the difference was not statistically significant.^35^ Conversely, in a small RCT of patients undergoing hepatic resection for cancer, Zhang and colleagues^36^ demonstrated that intraoperative infusion of dexmedetomidine did not have a clinically relevant effect on postoperative creatinine. Also, Wu and colleagues^37^ conducted an RCT in 40 patients undergoing abdominal surgery for biliary malignancies. The authors measured cystatin C, creatinine, blood urea nitrogen, and retinol-binding protein as biomarkers of renal function.^37^ Whilst dexmedetomidine improved renal function based on serum biomarkers compared with placebo, the differences were marginal and most likely not clinically relevant.^37^ Moon and colleagues^34^ conducted a retrospective study on 1207 patients undergoing lung cancer surgery and concluded that the administration of dexmedetomidine was not renoprotective. Lastly, the impact of dexmedetomidine (compared with placebo) on kidney function was investigated in patients (*n*=40) undergoing cytoreductive and intraperitoneal hyperthermic chemotherapy. Dexmedetomidine transiently and significantly reduced the serum concentration of neutrophil gelatinase-associated lipocalin and kidney injury molecule-1.^38^ It is worth mentioning that whilst the AKI rate based on the KDIGO definition was not statistically different, patients treated with dexmedetomidine showed an incidence of 11% compared with 26% in the placebo group.^38^

Our study also revealed other interesting findings. We observed an increase in postoperative complications, including cardiovascular and infectious events, in patients treated with dexmedetomidine as part of the general anaesthesia regimen. Patients who received dexmedetomidine had a longer duration of anaesthesia, which may be related to more complex surgical procedures. This may explain why patients in the dexmedetomidine group had higher rates of postoperative bleeding that required surgery and utilised more blood products (fresh frozen plasma and cryoprecipitate). Significantly, the administration of blood products has been associated with an increased risk of infection and cardiovascular events.^39^^,^^40^ Lastly, our multivariable analysis indicated that using vasopressors during surgery was associated with an increased risk of AKI. This finding is supported by a recent study, in which Chiu and colleagues^3^ demonstrated that intraoperative vasopressors during abdominal surgery were associated with an increased risk of postoperative AKI.

Our study has several limitations. The study results are likely biased by unmeasured confounding and confounding by indication (as anaesthesiologists decided which patients should receive dexmedetomidine and with which combinations of other drugs). Endpoint adjudication bias is a concern in retrospective studies. Although 30 day mortality was confirmed through a manual chart review, other complications were not. Second, residual imbalances exist between the groups in both baseline characteristics and intraoperative events likely to influence the risk of AKI. Matching patients by AKI score would be an alternative matching strategy in future studies. However, in this study, matching patients by AKI risk score alone was not performed because the score is not routinely available to anaesthesiologists who might have chosen preoperatively to administer dexmedetomidine based on the risk of AKI. Third, our database does not contain information on creatinine values beyond 7 days. Lastly, 30 day mortality, but no other complications were confirmed through the manual chart review. Therefore, this study did not address any potential impact of an anaesthetic regimen, including dexmedetomidine on persistent postoperative AKI.^41^ Fourth, the exclusion criteria in our study limit the generalisability of our findings. Lastly, our data do not contain information on continuous blood pressure or heart rate monitoring. Therefore, we cannot exclude the possibility that patients who received dexmedetomidine had significant episodes of hypotension or bradycardia during surgery.

In conclusion, the i.v. Administration of dexmedetomidine as part of a general anaesthetic regimen during major cancer surgery was not associated with a reduction in postoperative AKI. Because the literature remains ambiguous on the efficacy of dexmedetomidine to prevent AKI after major cancer surgery, a large RCT is needed.

## Authors’ contributions

Data collection: MAP-F

Data analysis: LF

Data interpretation: KM, JPC

Drafting of paper: MAP-F, JDL, TM, SB, KH, SH, NPC, MFR, KM, JPC

Final approval of paper: all authors.

## Declarations of interest

The authors declare that they have no conflicts of interest.
